# Investigating the efficacy of digital cognitive behavioural therapy in comparison to a sleep‐monitoring application via integrated diary and actigraphy: A randomised–controlled trial

**DOI:** 10.1111/jsr.14255

**Published:** 2024-06-19

**Authors:** Leonie Franziska Maurer, Pauline Bauermann, Lena Karner, Charlotte Müller, Noah Lorenz, Annika Gieselmann

**Affiliations:** ^1^ mementor DE GmbH Leipzig Germany; ^2^ Leipzig University Leipzig Germany; ^3^ University of Marburg Marburg Germany; ^4^ Friedrich Schiller University Jena Germany; ^5^ Heinrich Heine University Düsseldorf Germany

**Keywords:** cognitive behavioural therapy, digital therapeutics, mechanisms, self‐monitoring application, smartphone‐based therapy

## Abstract

Dissemination of digital cognitive behavioural therapy is a promising approach for treating insomnia in the broad population. Current evidence supports the effectiveness of the digital format, but clinical findings are often limited by the choice of control group and lack of in‐depth therapeutic measures. This study was designed to investigate the specific effects of digital cognitive behavioural therapy in comparison to a self‐monitoring application. Participants meeting criteria for insomnia were randomly allocated (1:1) to 8 weeks of digital cognitive behavioural therapy or 8 weeks of digital sleep monitoring (control application). The primary outcome, insomnia severity, was assessed at baseline, 8‐ and 16‐weeks post‐randomisation. Secondary outcomes included the assessment of sleep via application‐integrated sleep diaries and actigraphy. Linear‐mixed models were fitted to assess between‐group differences. Fifty‐six participants (48 females, mean age: M = 45.55 ± 13.70 years) were randomised to either digital cognitive behavioural therapy (*n* = 29) or digital sleep monitoring (*n* = 27). At 8‐ and 16‐weeks post‐randomisation, large treatment effects (*d* = 0.87–1.08) indicated robust reductions (−3.70 and −2.97, respectively; *p* ≤ 0.003) in insomnia severity in the digital cognitive behavioural therapy arm, relative to digital sleep monitoring. Treatment effects in favour of digital cognitive behavioural therapy were also found for self‐reported and actigraphy‐derived sleep continuity variables, indicating that sleep improved throughout the 8‐week intervention period. Our study reinforces the role of digital cognitive behavioural therapy in achieving clinical improvements for patients with insomnia, affirming previous findings and supporting the specific effects of cognitive behavioural therapy.

## 1 INTRODUCTION

Cognitive behavioural therapy for insomnia (CBT‐I) has been proven to be highly effective, and is recommended as the primary treatment for insomnia by major healthcare organisations worldwide (Edinger et al., 2021a; Riemann et al., 2023). This recommendation is based on a substantial body of evidence from well over 200 randomised–controlled trials (RCTs; Edinger et al., 2021b; van Straten et al., 2018). Despite the strong clinical evidence supporting its effectiveness, CBT‐I has faced limitations in accessibility (Thomas et al., 2016). To improve availability, various strategies have been suggested and employed, including delivering multi‐component CBT‐I through different formats such as group, telemedicine, bibliotherapy and digital interventions. Digital CBT‐I technologies have been developed across the globe over the past two decades, and its benefits are now substantiated by solid evidence showing that well‐designed digital CBT‐I interventions can effectively decrease insomnia severity, yielding effect sizes similar to those achieved through in‐person care (Soh et al., 2020; Zachariae et al., 2016). Moreover, standalone digital CBT‐I approaches have demonstrated enhancements in various daytime functions, encompassing improvements in anxiety, depression, fatigue, wellbeing and quality of life (Espie et al., 2019; Freeman et al., 2017; Lorenz et al., 2019; Schuffelen et al., 2023). Besides the advantages of digital CBT‐I with regards to time and costs, standalone digital CBT‐I may also present a suitable therapy format to test the specific factors related to the type of psychotherapeutic intervention in the absence of a therapist (Seiferth et al., 2023). Digital formats provide an opportunity to design *active* control groups that aim to resemble the unspecific functions of intervention applications (e.g. automated reminder and feedback), and thereby providing evidence for digital CBT‐I beyond non‐active (e.g. waitlist or treatment as usual) and minimally active (e.g. sleep hygiene education) control groups, which are currently the most commonly used control groups (Soh et al., 2020), and limit the validity of digital CBT‐I effectiveness data.

To test the specific effects of digital CBT‐I against an active comparator, we: (1) designed a personalised and fully automated control application, that was matched for self‐monitoring, a key feature of digital solutions, and also known as one of the unspecific treatment factors of psychotherapy by raising awareness for own behaviours and emotions, and thus facilitating self‐management (Bandura, 1998; Enck & Zipfel, 2019); and (2) embedded daily measures of sleep via integrated sleep diary and actigraphy throughout the intervention period in the trial design.

The primary aim of this study is to test whether digital CBT‐I (*somnio*, mementor DE GmbH, Leipzig, Germany) is effective in reducing insomnia symptoms compared with a specifically developed self‐monitoring application (digital sleep monitoring; *malio*, mementor DE GmbH, Leipzig, Germany). To present a full picture, we included further secondary outcomes that are: (1) known to be affected by insomnia (wellbeing, quality of life, depression and anxiety); and (2) assumed to be CBT‐I treatment mechanisms (dysfunctional beliefs about sleep and pre‐sleep arousal), although the study design may not be robust enough to test all secondary outcomes. The following primary and secondary hypotheses were tested.

1. Digital CBT‐I reduces self‐reported insomnia severity relative to control (assessed at 8 [primary endpoint] and 16 weeks).
2. Digital CBT‐I reduces wake‐time after sleep onset (WASO) and sleep‐onset latency (SOL), and increases sleep efficiency (SE) during the treatment phase (weeks 1–8) relative to control as indicated by sleep diary and actigraphy.
3. Digital CBT‐I reduces dysfunctional beliefs about sleep relative to control (assessed at 8 and 16 weeks).
4. Digital CBT‐I reduces pre‐sleep arousal relative to control (assessed at 8 and 16 weeks).
5. Digital CBT‐I improves wellbeing relative to control (assessed at 8 and 16 weeks).
6. Digital CBT‐I improves quality of life relative to control (assessed at 8 and 16 weeks).
7. Digital CBT‐I reduces self‐reported symptoms of depression and anxiety relative to control (assessed at 8 and 16 weeks).

## 2 METHODS

### 2.1 Study design and participants

The present trial was a randomised–controlled evaluation of digital CBT‐I versus digital sleep monitoring (control application). The intervention period lasted 8 weeks, and assessments took place at baseline, 8‐ and 16‐weeks post‐randomisation. The study was conducted in Leipzig, Germany, and approved by the Ethics Committee of the Heinrich Heine University Duesseldorf. It was preregistered at the German Clinical Trials Register (Deutsches Register Klinischer Studien; DRKS; https://drks.de) under DRKS00030897. The study protocol can be requested from the corresponding author.

Participants were recruited between January and July 2023 in Leipzig (Germany) and its surrounding area through community advertisement (posters and social media). General inclusion criteria were: aged ≥ 18 years, meeting criteria for DSM‐5 insomnia disorder (Insomnia Severity Index [ISI] ≥ 10 and diagnostic interview), being confident with smartphone/tablet/computer, and reliable access to the internet. Participants were excluded if they met one of the following criteria: current diagnosis for bipolar disorder, epilepsy, schizophrenia or acute psychosis, pregnancy, meeting criteria for other sleep disorders, regular consumption of alcohol (≥ 3 glasses daily for at least 3 weeks), use of cannabis (≥ 1 time per week), suicidal thoughts or intentions within the last 2 weeks, shift work, and current psychotherapy for sleep. We did not exclude for symptoms of anxiety or depression. Participants were not financially compensated for their participation, but received a visual and descriptive report on their actigraphy data upon completion of their follow‐up assessment.

The eligibility assessment process consisted of two stages. First, participants completed an online questionnaire using SoSci Survey 3.5.0 (SoSci Survey GmbH, Munich, Germany). Subsequently, a semi‐structured telephone interview was conducted to determine inclusion or exclusion. The telephone interview was undertaken by trained psychologists in their clinical psychotherapy master under supervision of a psychotherapist. Those found eligible were then invited for an in‐person study induction, where study procedures were discussed, written consent was obtained, and baseline assessments took place.

After the completion of baseline measurements, participants were randomised and scheduled for their in‐person post‐treatment assessment, and received a briefing for their actigraphy watch (MotionWatch 8, Camntech, Fenstanton, UK). Depending on the intervention they were allocated to, participants directly installed the assigned application (*somnio* or *malio*) with the study personnel. At 8‐weeks post‐randomisation, participants were invited for a second study visit to complete post‐treatment assessments and return their actigraphy watch. At 16‐weeks post‐randomisation, participants were provided with a link to an online follow‐up questionnaire using SoSci Survey.

Shortly after recruitment commenced, a change to the trial protocol was made and we included the opportunity for 15 randomly selected participants in the intervention group to take part in a semi‐structured interview at post‐treatment assessment, of which 13 took part. The results of this qualitative analysis will be reported separately. Additionally, the exclusion criteria “shift work” was added later to the registry, but included in the study protocol before recruitment commenced.

### 2.2 Randomisation and masking

Participants were assigned to either digital CBT‐I or self‐monitoring control in a 1:1 ratio using a randomisation sequence generated online (sealed envelope, London, UK). The randomisation was stratified based on age categories (18–39 versus ≥ 40 years), sex (female/male) and intake of prescribed sleep medication (yes/no), utilising varying block sizes (2–4) to ensure concealment of future allocations. The randomisation sequence was maintained by a researcher who had no contact with the participants and was not involved in any study procedures.

### 2.3 Interventions

Participants were informed that they would be randomly allocated to one of two digital sleep interventions, which are both considered advantageous for improving sleep but have not been compared in one study before. Participants in the digital CBT‐I group were given access to *somnio*. The *somnio* program is based on face‐to‐face CBT‐I manuals and consists of 10 core modules including information on psychoeducation, sleep restriction therapy (minimum time in bed [TIB] set to 6 hr), stimulus control, relaxation, and cognitive therapy, each requiring 5–25 min for completion. It operates on a fully automated platform and utilises an interactive and animated avatar to deliver the content. The application unlocks new content as participants complete previous modules and enter sleep diary data. Moreover, subsequent modules focus on relapse prevention and reinforce previously acquired knowledge (for detailed description, see Schuffelen et al., 2023). The clinical effects of *somnio* have already been confirmed in two RCTs (Lorenz et al., 2019; Schuffelen et al., 2023) and one retrospective user data analysis of its use in primary care (Maurer et al., 2023).

Participants in the control group were given access to *malio*, a digital sleep‐monitoring application specifically designed for the purpose of this trial. The *malio* program exclusively consists of the sleep diary feature included in *somnio*. It instructs participants to fill out a daily sleep diary in the morning and in the evening, and offers personalised descriptive and visual overviews of various sleep (SE, total sleep time [TST], SOL, WASO, TIB) and daytime variables (alcohol and caffeine consumption, mood, energy, and performance) over the past 7 days and since starting the program. The control application was fully automated and matched *somnio* in visuals and graphical features.

Both programs, *somnio* and *malio*, offer the possibility to activate regular reminders to continue with the program and to contact a support team for technical support within 24 hr. The intervention period was set up for 8 weeks, and both groups were told that they could receive access to the application they have not been assigned to upon completion of the 16‐weeks follow‐up assessment. Although both interventions were positioned as expected to be equally effective, credibility and expectancy of the interventions were not assessed.

### 2.4 Measures

#### 2.4.1 Primary outcome

Self‐reported insomnia severity was assessed using the German version of the ISI (Bastien et al., 2001; Dieck et al., 2018). This index examines sleep patterns over the preceding 2 weeks, and comprises seven items rated on a five‐point Likert scale, ranging from 0 (not at all) to 4 (extremely). Higher scores on the ISI indicate a higher level of insomnia severity. The individual item scores are combined to derive a total score within the range of 0–28. The ISI demonstrates good consistency, with a coefficient alpha of ≥ 0.90 (Morin et al., 2011).

#### 2.4.2 Secondary outcomes

##### Self‐reported sleep

Self‐reported sleep was obtained during the 8‐week intervention period with the integrated functions of *somnio* and *malio*, which consist of a morning and an evening log. The morning log asks about last night's sleep parameters, that is, SOL(= [“sleep onset” – “lights off”]), WASO (= [“time awake after sleep onset”] + [“rise time” – “wake‐up time”]), TST (= [“wake‐up time” – “sleep onset” – “time awake after sleep onset”]), TIB (= [rise time – bedtime]) and SE (= [TST/TIB × 100]). TST and TIB were analysed exploratively to provide a full picture of participants' sleep.

##### Objective sleep

Concurrently, participants wore an actigraph watch (MotionWatch 8, Camntech, UK) for the entire 8‐week duration of the intervention. The watch was configured to record activity and light in Motion Watch mode 1 in 60‐s epochs, aligning with recording settings commonly used in sleep studies (Ancoli‐Israel et al., 2003). Participants were instructed to press a designated marker button on the watch upon going to bed. These event markers were utilised to score sleep periods. In cases where event markers were not present, sleep periods were determined based on the sleep diary's recorded bed and rise times, or inferred from light and movement measurements according to a standardised, internal protocol. All sleep‐related parameters of interest were computed using the validated in‐built algorithm of the MotionWare software version 1.3.33.

##### Dysfunctional Beliefs and Attitudes about Sleep (DBAS)

We used the German 16‐item version of the DBAS scale to assess dysfunctional beliefs about sleep (Lang et al., 2017; Morin et al., 2007). Each item is rated from 0 (strongly disagree) to 10 (strongly agree). The average scores are combined to create an overall index, with higher scores indicating more pronounced dysfunctional beliefs and attitudes. The reliability of the German version is indicated by a Cronbach's alpha of *α* = 0.58–0.77 (Lang et al., 2017). However, due to an implementation error in SoSci survey, a 10‐point Likert scale (lacking 0) was used instead of an 11‐point Likert scale. In the current study, with the modified scaling, Cronbach's alpha was *α* = 0.87. For analysis, ratings were converted to a 0–10 scale ([*X* – 1] × [10/9]).

##### Pre‐sleep arousal

The Pre‐Sleep Arousal Scale (PSAS) is a self‐assessment questionnaire typically consisting of 16 items (Nicassio et al., 1985), yet the German version only consists of 15 items (Gieselmann et al., 2014). These items are divided into two categories: cognitive and somatic, each containing seven and eight items, respectively. Participants rate each item on a scale from 1 (not at all) to 5 (extremely). The total score for each category ranges from 7 to 35 and from 8 to 40, with higher scores indicating greater pre‐sleep arousal. The PSAS demonstrates strong internal consistency in both the cognitive and somatic categories when applied to individuals with insomnia (*α* ≥ 0.72; Nicassio et al., 1985).

##### Wellbeing

The World Health Organisation‐Five wellbeing index (WHO‐5; Brähler et al., 2007; Topp et al., 2015; WHO, 1998b) assesses mental wellbeing using five items on a six‐point Likert scale ranging from 0 (none of the time) to 5 (all of the time). The individual item scores are added together to form a total score and multiplied by 4, ranging from 0 to 100, where higher scores signify a higher level of wellbeing.

##### Quality of life

Self‐reported quality of life was measured using the brief version of the WHO quality of life questionnaire (WHOQOL‐BREF; WHO, 1998a). The questionnaire consists of 26 items, which are rated on a five‐point Likert scale from 1 to 5; it distinguishes between four quality of life domains: physical health, psychological health, social relationships, and environment. Domain scores are converted to a scale from 0 to 100, with higher scores indicating better quality of life. The internal consistency ranges from *α* = 0.66 to *α* = 0.84 across the domains (WHO, 1998a).

##### Symptoms of anxiety and depression

Symptoms of depression and anxiety were measured with the Hospital Anxiety and Depression Scale (HADS; Zigmond & Snaith, 1983). The questionnaire consists of 14 items (seven covering depressive symptoms and seven covering anxiety symptoms), which are rated from 0 to 3, yielding a sum score between 0 and 21, with higher scores indicating greater severity. The questionnaire reveals acceptable internal consistencies for both scales (anxiety: *α* = 0.68–0.93; depression: *α* = 0.67–0.90; Bjelland et al., 2002).

### 2.5 Statistical analyses

The sample size was calculated using G*Power (Faul et al., 2007) with *α* = 0.05 and power of 95%, to detect large effect sizes (*d* = 1.00) on the primary outcome at post‐treatment (week 8). The effect size calculation was based on digital CBT‐I trials with active control conditions that were derived from a meta‐analysis on digital CBT‐I (Soh et al., 2020). Power‐analysis revealed a sample size of *N* = 56 participants, which would be needed to detect between‐group effects. Accounting for 10% attrition, we sought to recruit 60 participants (30 in each group).

Available data from all randomised participants were analysed according to the intention to treat principle (Hollis & Campbell, 1999). For between‐group comparisons of the primary outcome, a linear‐mixed‐effects regression model was fitted with fixed effects of group and time point and accounted for missing data. The outcomes at 8 and 16 weeks were included as the response. To adjust for baseline measures, the baseline score was entered as a covariate and a participant‐specific random intercept was added to account for repeated measures (Landau & Everitt, 2017). An interaction between time point and group was included to estimate treatment effects at each time point. The covariance structure was set to unstructured. Cohen's *d* statistics were calculated as the adjusted treatment effect divided by the baseline standard deviation of the outcome for the combined groups (Cohen, 1988). Secondary outcomes were analysed in the same way. For continuous sleep diary variables and in line with recommendations for standard research assessments of insomnia (Buysse et al., 2006), responses were entered as averages of 2‐week periods and compared with late treatment (weeks 7–8), adjusting for values at the beginning of the treatment (weeks 1–2). Summary statistics are presented in the form of unadjusted means and standard deviations. All analyses were conducted in SPSS.29 (IBM).

## 3 RESULTS

**FIGURE 1:**
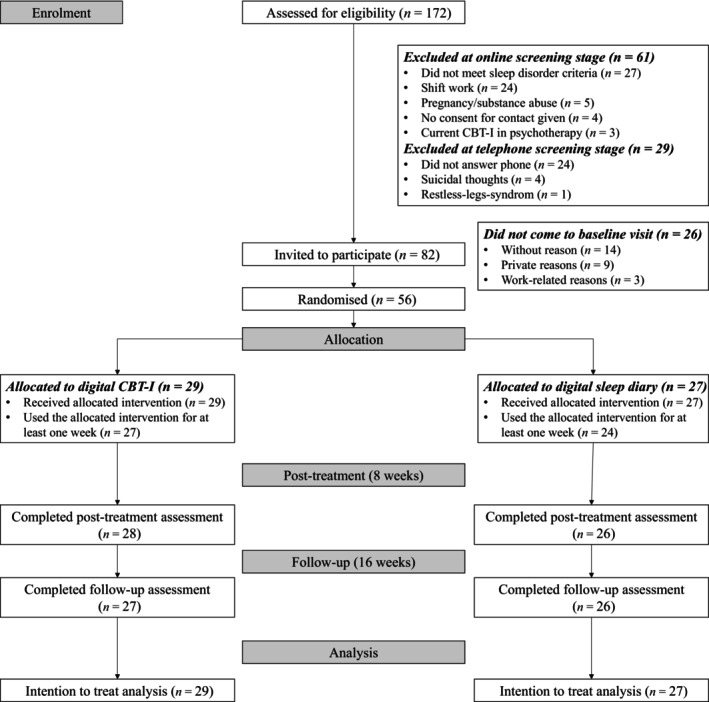
Participant recruitment Consolidated Standards of Reporting Trials (CONSORT)‐style flow diagram.

Overall, *N* = 172 potential participants completed the screening between December 2022 and July 2023. Of these, *N* = 109 fulfilled requirements for further participation and provided contact information for the telephone screening. Main reasons for exclusion were shift work and not meeting insomnia criteria. After the clinical interview, *N* = 81 participants were still interested in participating, deemed eligible, and invited for their in‐person baseline visit. Twenty‐five of those invited did not show up for their study visit, resulting in *N* = 56 participants, who completed all baseline assessments and were subsequently randomised. None of the participants actively withdrew from the study or the intervention; however, five participants used the allocated intervention for less than 1 week (digital CBT‐I: *N* = 2; digital sleep diary: *N* = 3) and three participants were marked as lost‐to follow‐up at week 16 (digital CBT‐I: *N* = 2; digital sleep diary: *N* = 1). The participant flow is shown in Figure 1 (CONSORT flow diagram).

The majority of the participants were middle‐aged, with an average age of 45.55 ± 13.70 years. The sample primarily consisted of women (86%), and most of the participants reported having received a university education (52%). In line with the specified criteria for inclusion, the average ISI score of 16.66 ± 3.41 across all participants indicated clinical levels of insomnia. Moreover, subscales of the HADS showed moderate levels of anxiety and mild levels of depression symptoms. Notably, the two groups were closely aligned in terms of demographic factors and clinical outcomes at the start of the study (Table 1). Post‐treatment assessments took place from March 2023 to August 2023 (post‐treatment), and May 2023 to November 2023 (follow‐up).

**TABLE 1:** Participant characteristics by group.

	Digital CBT‐I		Digital sleep diary	
	*N* = 29		*N* = 27	
Baseline characteristics				
Age, years, M (SD)	45.21	12.30	45.93	15.27
Female, *N* (%)	24	82.80	24	88.90
Symptom duration, years, M (SD)	7.76	7.04	7.91	6.68
Highest level of qualification, *N* (%)				
Middle school	1	3.40	1	3.70
High school degree	2	6.90	2	7.40
Apprenticeship	13	44.80	8	29.60
University degree	13	44.80	16	59.30
Psychotherapy				
Current, *N* (%)	3	10.30	4	14.80
Former, *N* (%)	8	27.60	10	37.00
Medication				
CNS medication, *N* (%)	6	20.70	3	11.10
Sleep medication, *N* (%)	5	17.20	2	7.40
Other medication, *N* (%)	17	58.60	9	33.30
Comorbidities				
Acute mental illness, *N* (%)	5	17.20	5	18.50
Past mental illness, *N* (%)	5	17.20	5	18.50
Coronary heart disease, *N* (%)	7	24.10	2	7.40
Pulmonary disease, *N* (%)	4	13.80	0	0.00
Gastrointestinal disease, *N* (%)	2	6.90	1	3.70
Endocrine disease, *N* (%)	8	27.60	7	25.90
Neurological disease, *N* (%)	3	10.30	2	7.40
Cancer, *N* (%)	0	0.00	1	3.70
Chronic pain, *N* (%)	3	10.30	4	14.80
Polyneuropathy, *N* (%)	2	6.90	1	3.70
Outcomes at baseline				
ISI, M (SD)	16.90	2.90	16.41	3.96
DBAS, M (SD)	82.03	24.77	76.87	29.98
PSAS				
Cognitive subscale, M (SD)	21.41	5.79	17.41	6.24
Somatic subscale, M (SD)	15.59	4.44	14.07	5.69
WHO‐5 wellbeing index, M (SD)	36.83	18.12	43.56	21.15
WHOQOL‐BREF				
Physical health, M (SD)	31.40	7.17	34.13	6.74
Psychological health, M (SD)	57.90	11.54	59.72	14.06
Social relationships, M (SD)	64.94	24.44	61.42	18.80
Environment, M (SD)	76.29	12.18	75.46	12.31
HADS				
Anxiety subscale, M (SD)	11.83	1.81	12.11	1.78
Depression subscale, M (SD)	9.72	1.36	10.07	1.77

*Note*: In the absence of baseline data, sleep variables at the beginning of the intervention are presented in Table 3.

Abbreviations: CBT‐I, cognitive behavioural therapy for insomnia; CNS, central nervous system; DBAS, Dysfunctional Beliefs and Attitudes about Sleep; HADS, Hospital Anxiety and Depression Scale; ISI, Insomnia Severity Index; M, means; *N*, number of participants; PSAS, Pre‐Sleep Arousal Scale; SD, standard deviations; WHO‐5, World Health Organisation‐Five; WHOQOL‐BREF, brief version of WHO quality of life questionnaire.

### 3.1 Primary outcome

#### 3.1.1 Insomnia Severity Index

**FIGURE 2:**
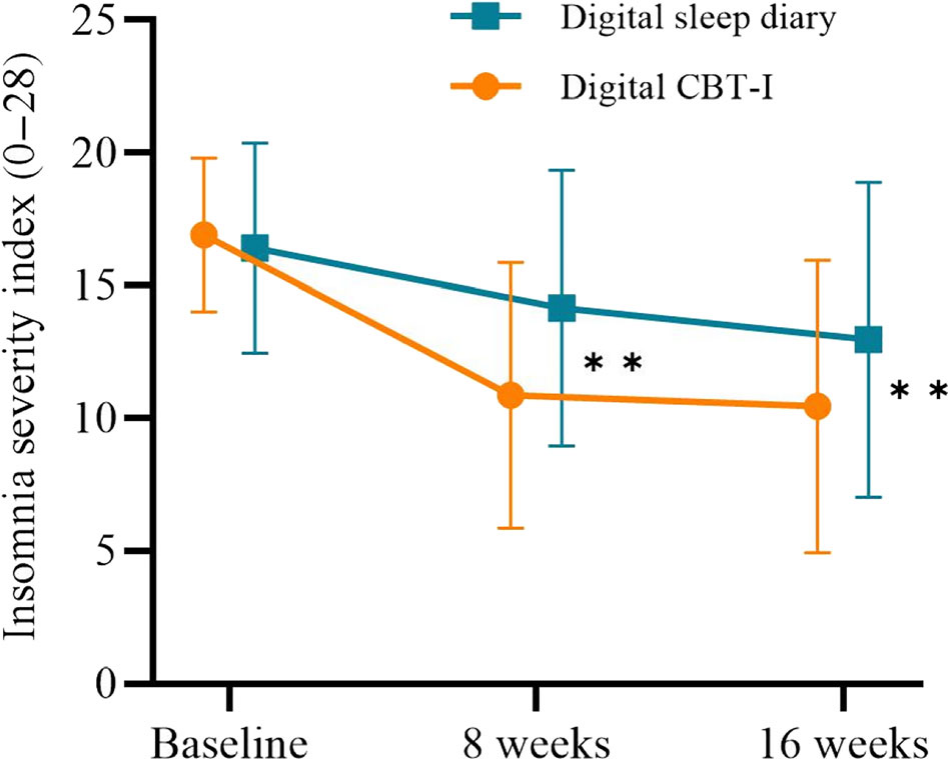
Changes in the primary outcome, insomnia severity, across groups and time points. Raw means (± 1 SD) are presented for both groups at each time point. Statistical group differences are derived from linear‐mixed models and represented by a double asterisk (**p < 0.010).

Our primary hypothesis was to assess if digital CBT‐I reduces self‐reported insomnia severity relative to digital sleep monitoring. Between‐group comparisons revealed large treatment effects at 8 (*p* < 0.001; *d* = 1.08) and 16 weeks (*p* = 0.003, *d* = 0.87) in favour of the digital CBT‐I group. On average, ISI scores were reduced by 3.70 and 2.97 points, in comparison to digital sleep monitoring (Figure 2; Table 2).

**TABLE 2:** Effects of digital CBT‐I versus digital sleep monitoring on clinical outcomes.

Outcome	Digital CBT‐I		Digital sleep diary		Diff_adj_	*p*	95% CI		
	M	SD	M	SD					ES
Sleep diary									
ISI									
Week 8	10.86	5.00	14.15	5.20	−3.70	**< 0.001**	−5.63	−1.76	−1.08
Week 16	10.44	5.51	12.96	5.93	−2.97	**0.003**	−4.92	−1.02	−0.87
DBAS									
Week 8	59.52	31.64	76.79	28.30	−19.68	**< 0.001**	−29.68	−9.68	−0.73
Week 16	61.44	34.96	70.43	32.82	−14.65	**0.005**	−24.74	−4.56	−0.55
Cognitive pre‐sleep arousal									
Week 8	17.11	5.71	14.38	5.50	−3.71	**0.004**	−6.22	−1.21	−0.62
Week 16	16.67	6.05	14.50	5.24	−2.44	0.058	−4.97	0.09	−0.41
Somatic pre‐sleep arousal									
Week 8	13.25	5.12	14.38	5.50	−1.93	0.086	−4.13	0.28	−0.38
Week 16	14.26	6.10	14.50	5.24	−1.23	0.277	−3.45	1.00	−0.24
Wellbeing									
Week 8	47.43	23.84	46.46	23.92	6.24	0.194	−3.20	15.68	0.32
Week 16	49.93	23.54	44.46	24.22	10.57	**0.030**	1.06	20.08	0.54
Quality of life: Psychological health									
Week 8	61.61	11.75	58.81	12.71	4.79	**0.027**	0.54	9.04	0.37
Week 16	59.41	13.46	55.93	15.51	5.05	**0.021**	0.77	9.33	0.39
Quality of life: Physical health									
Week 8	37.37	8.28	38.32	7.80	1.03	0.535	−2.24	4.30	0.15
Week 16	37.70	7.17	38.46	7.91	0.67	0.690	−2.62	3.95	0.10
Quality of life: Social relationships									
Week 8	68.75	23.53	59.29	16.04	6.01	**0.048**	0.05	11.96	0.28
Week 16	62.96	21.97	60.90	20.38	−0.18	0.953	−6.18	5.83	−0.01
Quality of life: Environment									
Week 8	75.11	13.30	73.32	11.67	0.63	0.757	−3.36	4.61	0.05
Week 16	72.80	15.08	69.83	17.32	2.21	0.278	−1.80	6.23	0.18
Symptoms of anxiety									
Week 8	12.39	1.73	12.50	1.94	0.02	0.963	−0.76	0.80	0.01
Week 16	12.48	1.58	12.77	2.49	−0.16	0.692	−0.95	0.63	−0.09
Symptoms of depression									
Week 8	9.96	1.50	9.54	1.48	0.63	0.065	−0.04	1.30	0.40
Week 16	9.89	1.40	10.54	2.00	−0.37	0.287	−1.05	0.31	−0.23

*Note*: Significant *p*‐values are displayed in bold.

Abbreviations: CBT‐I, cognitive behavioural therapy for insomnia; 95% CI, 95% confidence interval of the adjusted mean difference; DBAS, Dysfunctional Beliefs and Attitudes about Sleep; Diff_adj_, adjusted mean difference derived from linear‐mixed model; ES, effect size (Cohen's *d*); ISI, Insomnia Severity Index; M, unadjusted means; *N*, number of complete data sets; SD, standard deviations.

### 3.2 Secondary outcomes

#### 3.2.1 Self‐reported sleep

Sleep diary data were available for 51 participants (digital CBT‐I: *N* = 27; digital sleep diary: *N* = 24), who recorded on average 49.06 ± 15.52 daily diary entries during the 8 weeks of intervention (digital CBT‐I: *N* = 51.15 ± 13.45; digital sleep diary: *N* = 46.71 ± 17.26). Between‐group comparisons at the end of treatment (weeks 7–8) revealed small‐to‐medium effects in favour of the digital CBT‐I group across sleep continuity outcomes (*d* = 0.25–0.37): SOL (−18.45 min, *p* < 0.001), WASO (−22.23 min, *p* < 0.001), SE (+7.38%, *p* < 0.001). Additionally, TIB was reduced by −25.93 min, when compared with digital sleep monitoring (*p* = 0.007). No between‐group differences were found for TST (+12.57 min, *p* = 0.176). Changes in sleep continuity variables are also shown in Figure S1.

#### 3.2.2 Actigraphy‐derived sleep

Actigraphy data were available for 52 participants (digital CBT‐I: *N* = 27; digital sleep diary: *N* = 25). On average, sleep data were available for 45.13 ± 14.75 nights (digital CBT‐I: M = 48.15 ± 11.41; digital sleep diary: M = 41.88 ± 17.08). Between‐group comparisons of sleep variables mostly reflected the results reported for self‐reported sleep, yet to a smaller degree (*d* = 0.21–0.29): SOL (−5.99 min, *p* = 0.010), WASO (−7.42 min, *p* = 0.040), SE (+1.97%, *p* < 0.001). Additionally, TIB was reduced by −25.31 min (*p* = 0.010), when compared with digital sleep monitoring. Again, no between‐group effects were found for TST (−9.80 min, *p* = 0.200). An overview of all sleep variables, self‐reported and actigraphy‐derived, is displayed in Table 3.

**TABLE 3:** Overview of self‐reported and actigraphy‐derived sleep variables.

Outcome	Digital CBT‐I		Digital sleep diary						
	M	SD	M	SD	Diff_adj_	*p*	95% CI		ES
Sleep diary									
SOL									
Weeks 1–2	46.55	46.82	55.14	65.29					
Weeks 7–8	27.69	32.36	59.77	65.58	−18.45	**< 0.001**	−27.33	−9.57	−0.33
WASO									
Weeks 1–2	90.70	69.79	118.19	109.21					
Weeks 7–8	55.49	49.77	95.45	67.69	−22.23	**< 0.001**	−36.75	−7.70	−0.25
SE									
Weeks 1–2	73.68	15.67	67.37	24.57					
Weeks 7–8	82.72	12.94	71.23	18.61	7.38	**< 0.001**	4.12	10.65	0.37
TST									
Weeks 1–2	380.80	98.76	345.81	130.10					
Weeks 7–8	394.57	100.16	371.53	106.49	12.57	0.176	−5.68	30.82	0.11
TIB									
Weeks 1–2	518.05	87.00	519.14	92.97					
Weeks 7–8	477.76	99.67	526.75	92.93	−25.93	**0.007**	−44.53	−7.32	−0.29
Actigraphy									
SOL									
Weeks 1–2	15.46	23.32	12.56	21.48					
Weeks 7–8	10.13	17.32	13.53	20.56	−5.99	**0.006**	−10.26	−1.71	−0.27
WASO									
Weeks 1–2	80.04	31.75	85.01	38.26					
Weeks 7–8	76.95	35.78	89.20	43.18	−7.42	**0.039**	−14.47	−0.37	−0.21
SE									
Weeks 1–2	80.60	7.59	80.00	7.66					
Weeks 7–8	81.20	7.19	78.90	7.58	1.97	**< 0.001**	0.67	3.26	0.26
TST									
Weeks 1–2	412.52	76.99	406.36	75.53					
Weeks 7–8	393.58	76.32	409.89	71.26	−9.80	0.195	−24.68	5.07	−0.13
TIB									
Weeks 1–2	512.36	85.60	509.50	90.99					
Weeks 7–8	485.77	90.00	521.60	91.24	−25.31	**0.010**	−44.57	−6.05	−0.29

*Note*: Significant *p‐*values are displayed in bold.

Abbreviations: CBT‐I, cognitive behavioural therapy for insomnia; 95% CI, 95% confidence interval of the adjusted mean difference; Diff_adj_, adjusted mean difference derived from linear‐mixed models; ES, effect size (Cohen's *d*); M, unadjusted means; SD, standard deviations; SE, sleep efficiency; SOL, sleep‐onset latency; TIB, time in bed; TST, total sleep time; WASO, wake‐time after sleep onset.

#### 3.2.3 Dysfunctional Beliefs and Attitudes about Sleep

Results from the linear‐mixed model revealed medium‐to‐large effect sizes at 8‐ (*p* < 0.001, *d* = 0.73) and 16‐weeks post‐randomisation (*p* = 0.005, *d* = 0.55; Table 2) in favour of the digital CBT‐I group, indicating that dysfunctional beliefs decreased as a result of the digital CBT‐I intervention.

#### 3.2.4 Pre‐sleep arousal

Between‐group comparisons showed medium treatment effects at 8‐weeks post‐randomisation in favour of the digital CBT‐I group for the cognitive PSAS scale (*p* = 0.004, *d* = 0.62). These effects were not maintained at 16‐weeks follow‐up, although effect sizes indicate a trend in the same direction (*p* = 0.058, *d* = 0.41). No between‐group effects were found for somatic pre‐sleep arousal at either time point (*p* ≥ 0.086; Table 2).

#### 3.2.5 Wellbeing

Analysis of overall wellbeing indicated medium treatment effects favouring the digital CBT‐I group at follow‐up when compared with digital sleep diary (*p* = 0.030, *d* = 0.54; Table 2). There were no between‐group effects at 8‐weeks post‐randomisation (*p* = 0.194, *d* = 0.32).

#### 3.2.6 Quality of life

Of the four subscales of the WHO‐5 quality of life measure, between‐group treatment effects were only found for the domains psychological health at post‐treatment (*p* = 0.027, *d* = 0.37) and follow‐up (*p* = 0.021, *d* = 0.39), and social relationships at post‐treatment (*p* = 0.048, *d* = 0.28). There were no between‐group effects for the domains physical health and environment at either time point (*p* ≥ 0.278; Table 2).

#### 3.2.7 Symptoms of anxiety and depression

There were no between‐group effects for symptoms of depression or anxiety at post‐treatment or follow‐up (*p* ≥ 0.065; Table 2). An overview of all treatment effects for questionnaire derived outcomes is shown in Figure S2.

#### 3.2.8 Adverse events

One person in the digital CBT‐I group reported stopping the intervention and wearing the actigraphy watch after 1 month, because it was too much effort and she felt distressed reminding herself about it.

## 4 DISCUSSION

The aim of this study was to investigate the efficacy of digital CBT‐I in comparison to an active comparator, realised with a digital sleep‐monitoring app, across measures of insomnia, self‐reported and actigraphy‐derived sleep variables, and clinical outcomes assessing wellbeing beyond the symptoms of insomnia. It was hypothesised that digital CBT‐I would be superior to simply monitoring sleep, a feature and function of digital CBT‐I, but also an unspecific treatment factor of psychotherapy. The superiority of digital CBT‐I was demonstrated by large treatment effects in our primary outcome, insomnia severity, at both follow‐up time points compared with the self‐monitoring application. Indeed, the effect size at 8 weeks (*d* = 1.08) was comparable to other digital CBT‐I trials using active control groups as calculated by our initial power analysis (*d* = 1.00). Further confirming our hypotheses, the digital CBT‐I arm reported a decrease in SOL and WASO, leading to improved SE compared with the digital sleep diary arm. Importantly, these sleep diary‐derived observations were supported by small effect sizes across sleep continuity metrics derived from actigraphy.

Interestingly, and although not formulated as a hypothesis and thereby explorative, analysis of TIB showed reductions of −25 min in the digital CBT‐I versus sleep‐monitoring arm, potentially reflecting sleep behaviour changes due to sleep restriction therapy (Maurer et al., 2018, 2021). Support for potential mechanisms‐of‐actions besides changes in sleep were also found for dysfunctional beliefs about sleep and cognitive pre‐sleep arousal, which were found to be reduced in the digital CBT‐I arm, when compared with digital sleep monitoring, and to date seem to have the greatest evidence as potential treatment mediators (Parsons et al., 2021).

Yet, mixed results were found for secondary clinical outcomes that measured outcomes beyond improving insomnia symptoms: (1) overall wellbeing improved at 16‐weeks follow‐up; (2) the quality of life domain psychological health was rated higher at both time points by the digital CBT‐I arm; (3) the quality of life domain social relationships was rated higher at 8‐weeks post‐treatment. Yet, no between‐group effects were reported for symptoms of anxiety and depression, physical health, and the quality of life domain environment. While mixed effects for quality of life have been reported previously, and are not fully supported by studies investigating online CBT‐I interventions (Alimoradi et al., 2022), small‐to‐medium effects on measures of anxiety and depression are typically observed as a result of internet‐based CBT‐I (Ye et al., 2016).

The primary strength of our study lies in the study design we employed. Our choice of an active control group, matched for sleep monitoring but not for the specific active components of CBT‐I, aligns with recent trends in psychotherapy research and recommendations for evaluating digital interventions to meticulously select and justify control conditions (Leichsenring et al., 2019; Michie et al., 2017). This approach in combination with the use of a digital format of delivering therapy empowers us to draw robust conclusions regarding the distinct impact of the CBT‐I intervention (e.g. no *therapist effect*). Additionally, our use of high‐resolution sleep measurements through diary and actigraphy, with consistent findings across these methods, adds credibility to our conclusions.

Considerations must be given to the limitations of this study. Our sample was exclusively drawn from the confined region of Leipzig, Germany, and the study protocol required high motivation. Consequently, the sample may not be a true representation of the broader insomnia population in Germany. It is important to acknowledge, however, that the primary objective of this study was not to assess generalisability but rather to evaluate the efficacy of digital CBT‐I in a self‐monitoring‐controlled setting. The digital sleep‐monitoring app, *malio*, was carefully designed, matched the functions and visuals of the integrated sleep diary function of *somnio*, and was introduced as an effective sleep intervention to the participants; however, we did not assess credibility and expectancy of participants when assigned to the intervention, which may have affected treatment participation and engagement. While rates of no usage of the sleep diary functions were comparable (digital CBT‐I: *N* = 2; digital sleep diary: *N* = 3), numbers of recordings differed slightly with on average 4 days less in the digital sleep‐monitoring arm, indicating that engagement may have been slightly lower. Additionally, it is important to mention that baseline values of the HADS showed moderate levels of anxiety and mild levels of depression symptoms. While this is a common observation in an insomnia population, we cannot determine how these symptoms may have affected our study endpoints. However, recent secondary analyses on subgroups of anxiety and depression using the same CBT‐I intervention suggested comparable treatment effects (Rötger et al., 2024). Lastly, the small sample size and the absence of baseline values for sleep diary and actigraphy data are limitations of our study design. Given the small sample size, the study was not powered to detect small‐to‐medium effect sizes and, consequently, a higher risk for a type II error was present for the analysis of some of our secondary outcomes. Moreover, in the absence of baseline data, post‐treatment comparisons of sleep diary and actigraphy outcomes were adjusted for weeks 1–2 of treatment to control for starting values. While typically no active CBT‐I components are introduced at this stage, data may have been influenced by group allocation, and thereby affected the adjustment.

## 5 CONCLUSION

Our study highlights the significance of digital CBT‐I in achieving clinical improvements, affirming findings from previous studies investigating the same intervention, and meta‐analyses on digital CBT‐I interventions. Moreover, our results support the efficacy of the specific CBT‐I components, yielding treatment effects in the absence of a therapist, and in comparison to self‐monitoring.

## AUTHOR CONTRIBUTIONS

**Leonie Franziska Maurer:** Conceptualization; investigation; writing – original draft; methodology; writing – review and editing; visualization; formal analysis; project administration; supervision; validation; data curation. **Pauline Bauermann:** Conceptualization; investigation; writing – review and editing; methodology. **Lena Karner:** Conceptualization; investigation; methodology; writing – review and editing. **Charlotte Müller:** Investigation; writing – review and editing. **Noah Lorenz:** Conceptualization; funding acquisition; writing – review and editing; resources. **Annika Gieselmann:** Conceptualization; investigation; writing – review and editing; project administration; supervision; methodology; resources.

## FUNDING INFORMATION

This research study was supported by the mementor DE GmbH. The views expressed are those of the authors and not necessarily those of the mementor DE GmbH.

## CONFLICT OF INTEREST STATEMENT

LFM and CM are salaried employees of the mementor DE GmbH, a company that specialises in digital health applications, one of which addresses the delivery of cognitive behavioural therapy for sleep improvement (*somnio*). NL is founder and salaried employee of mementor DE GmbH. The presented work is related to the named product (*somnio*). AG reports *non‐financial support* from the mementor DE GmbH in the form of no‐cost access to *somnio* for use in clinical trial research. All other investigators report no competing interests.

## Supporting information


**FIGURE S1.** Changes in sleep continuity variables, across groups and time points. Raw means (± 1 sleep efficiency [SE]) are presented for both groups at each time point. Statistical group differences at weeks 7–8 are derived from linear‐mixed models and represented by a single (*p < 0.050) or double asterisk (**p < 0.010). Graphs on the left‐hand side represent sleep continuity variables derived from sleep diaries. Graphs on the right‐hand side represent sleep continuity variables derived from actigraphy. Note that *y*‐scales were adjusted to allow the visible representation of all SEs.
**FIGURE S2.** Treatment effects across primary and secondary outcomes. Standardised effect sizes (SMD) are represented with 95% confidence intervals (whiskers) for each time point (8 and 16 weeks). DBAS, Dysfunctional Beliefs and Attitudes about Sleep; HADS, Hospital Anxiety and Depression Scale; ISI, Insomnia Severity Index; PSAS, Pre‐Sleep Arousal Scale; QOL, quality of life.

## Data Availability

The data that support the findings of this study are available on request from the corresponding author. The data are not publicly available due to privacy or ethical restrictions.
