# Supplementary material for: Investigating the efficacy of digital cognitive behavioural therapy in comparison to a sleep‐monitoring application via integrated diary and actigraphy: A randomised–controlled trial
Source: J Sleep Res. 2024 Jun 19;34(1):e14255. doi: 10.1111/jsr.14255 (PMC11744233; doi:10.1111/jsr.14255)
Supplement: Supplementary file 1 — FIGURE S1. Changes in sleep continuity variables, across groups and time points. Raw means (± 1 sleep efficiency [SE]) are presented for both groups at each time point. Statistical group differences at weeks 7–8 are derived from linear‐mixed models and represented by a single (*p < 0.050) or double asterisk (**p < 0.010). Graphs on the left‐hand side represent sleep continuity variables derived from sleep diaries. Graphs on the right‐hand side represent sleep continuity variables derived from actigraphy. Note that y‐scales were adjusted to allow the visible representation of all SEs. FIGURE S2. Treatment effects across primary and secondary outcomes. Standardised effect sizes (SMD) are represented with 95% confidence intervals (whiskers) for each time point (8 and 16 weeks). DBAS, Dysfunctional Beliefs and Attitudes about Sleep; HADS, Hospital Anxiety and Depression Scale; ISI, Insomnia Severity Index; PSAS, Pre‐Sleep Arousal Scale; QOL, quality of life. [file JSR-34-e14255-s001.docx]

# Supplementary


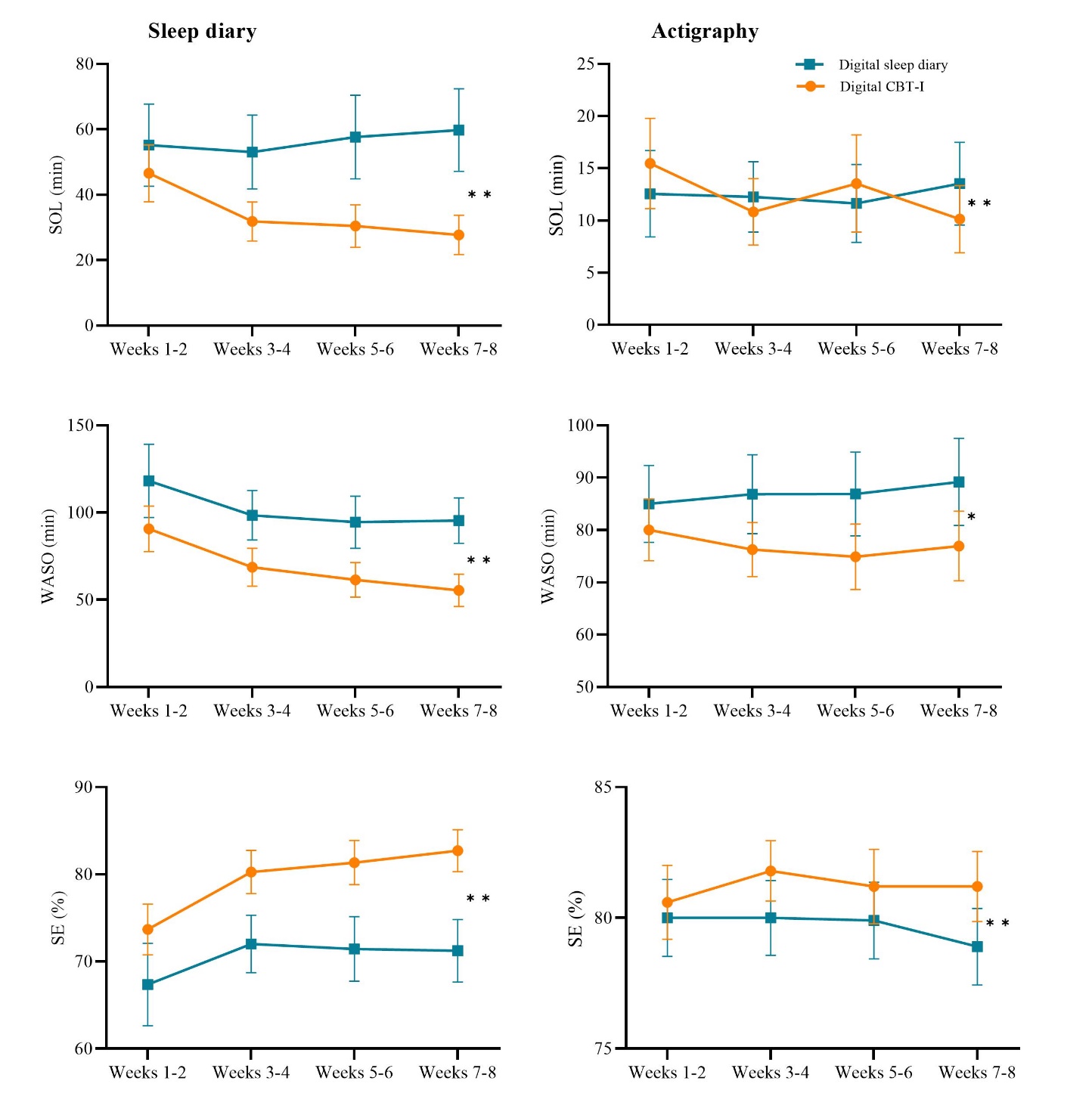


Figure S1. Changes in sleep continuity variables, across groups and time points. Raw means (+/− 1 SE) are presented for both groups at each time point. Statistical group differences at weeks 7−8 are derived from linear mixed models and represented by a single (**p* < .050) or double asterisk (***p* < .010). Graphs on the left-hand side represent sleep continuity variables derived from sleep diaries. Graphs on the right-hand side represent sleep continuity variables derived from actigraphy. Note that y-scales were adjusted to allow the visible representation of all *SE*s.

Figure S2. Treatment effects across primary and secondary outcomes. Standardised effect sizes (SMD) are represented with 95% confidence intervals (whiskers) for each time point (8 and 16 weeks). ISI, Insomnia severity index; DBAS, Dysfunctional beliefs about sleep; PSAS, Pre-sleep arousal scale; QOL, Quality of life; HADS, Hospital anxiety and depression scale
